# Effect of macular vascular density on visual quality in young myopic adults

**DOI:** 10.3389/fmed.2022.950731

**Published:** 2022-08-16

**Authors:** Xueqing Wang, Likun Xia

**Affiliations:** Shengjing Hospital of China Medical University, Shenyang, China

**Keywords:** myopia, vascular density, optical coherence tomography angiography, visual quality, contrast sensitivity

## Abstract

**Objective:**

To evaluate macular vascular density using optical coherence tomography angiography (OCTA) and to investigate its impact on best-corrected visual acuity (BCVA), contrast sensitivity function (CSF), and higher-order aberrations (HOAs) in young myopic adults.

**Methods:**

This cross-sectional study included 109 eyes with axial length (AL) between 22 and 26 mm in the medium AL group and 90 eyes with AL > 26 mm in the long AL group. OCTA was used to obtain 3 × 3 mm en face images, and the vessel length density (VLD), perfusion density (PD), and fovea avascular zone (FAZ) of the superficial layer were evaluated. Visual quality was assessed using the CSF and HOAs.

**Results:**

Significant differences were found in the inferior VLD, parafoveal PD, and FAZ areas between the groups. AL and macular vascular density showed negative correlations in the inferior and nasal areas. The spherical correction (SE) also showed a positive correlation with vascular density in these two areas. FAZ area and perimeter had a significant negative association with AL, and FAZ circularity was correlated with SE. CSF with bright around 6 and 12 spatial frequencies showed positive correlations with nasal PD. The parafoveal PD showed a significant correlation with BCVA after adjusting for other factors.

**Conclusion:**

The superficial macular vascular density of young myopic adults decreased with lower SE and longer AL in the parafovea area. An eye with a long AL has a smaller FAZ, and myopia decreases the FAZ circularity index. The decrease in vessel density could contribute to worse BCVA and may be correlated with lower CSF, but not with HOAs.

## Introduction

Myopia is one of the most common diseases worldwide, particularly in Asia (1). It is necessary to address myopia in the younger age groups as there is a decreasing trend in the age of patients acquiring myopia (2). High myopia, an extreme form of myopia, can cause sequelae such as myopic choroidal neovascularization and myopic macular degeneration, which may lead to blindness (1, 3). Pathological myopia, also known as degenerative myopia, is caused by high axial myopia with characteristic pathological changes (4). Therefore, the observation of a fundus with high myopia, especially in the macular area, the most sensitive area of vision, is essential for the early detection and diagnosis of retinal diseases (5).

In myopia-related retinal disorders, more attention should be given to changes in the retinal microvascular system, because it is a vital source of oxygen and nutrients (6). Optical coherence tomography angiography (OCTA) may help in capturing the early state of fundus lesions with alterations in retinal vessel morphology. It is a recently developed imaging technique that can obtain high-resolution fundus retinal imaging and quantitative microvascular information of multiple layers in a non-invasive and quick manner (7), and has been proven to have good reproducibility and reliability (8).

Previous studies have found that the decrease in retinal vessel density in high myopia may be caused by increased axial length (AL) (9, 10). Some studies have demonstrated that macular flow density affects best-corrected visual acuity (BCVA) (11), but others indicate otherwise (12); therefore, it is still a matter of controversy. The contrast sensitivity function (CSF) and higher-order aberrations (HOAs) are commonly used to evaluate visual (13). Previous studies have found that the visual quality decreases in patients with high myopia (14). We hypothesized that the reduced CSF in myopic patients is related to changes in fundus structure, and there are only a few studies that have discussed the correlation between visual quality and retinal vessel density in young myopes (11, 15).

This study aimed to investigate the distribution characteristics of superficial macular retinal vascular density and foveal avascular zone (FAZ) in young adults with myopia using OCTA, and to explore the correlation between retinal vascular density and visual quality, including CSF and HOAs.

## Materials and methods

### Subjects

A total of 199 eyes of 109 healthy myopic subjects (58 men and 51 women) were enrolled in this cross-sectional study between May 2019 and May 2020. This study was approved by the ethics committee of Shengjing Hospital, China Medical University, Shenyang, China, and followed the tenets of the Declaration of Helsinki. The participants understood the study protocol and provided signed informed consent.

All subjects underwent a full ophthalmic examination that included evaluation and measurement of uncorrected visual acuity (UCVA) and BCVA; refractive status assessment using an automatic refractometer (Auto Refractometer AR-1; NIDEK, Co., Ltd., Gamagori, Japan); intraocular pressure (IOP) measurement using a non-contact tonometer (KOWA KT-800; Kowa, Co., Ltd., Tokyo, Japan); anterior segment using a slit-lamp-assisted biomicroscope (Carl Zeiss Meditec, Germany); fundus using a digital retinal camera (CR-2; Canon, Tokyo, Japan); wavefront aberration for a 4-mm pupil diameter measured by the WASCA wavefront analyzer (Carl Zeiss Meditec AG, Jena, Germany); CSF using the Functional Vision Analyzer (Stereo Optical Co., Chicago, IL, United States) at five spatial frequencies; and AL measurement using the IOL Master (Carl Zeiss Meditec). BCVA and UCVA were measured using an international standard visual acuity chart at a distance of 5 m, and the logarithmic minimum angle of resolution (logMAR) was recorded. Mean spherical correction (SE) was calculated using the spherical diopter plus half of the cylindrical diopter.

The enrolled eyes were divided into a medium AL group (22 mm ≤ AL < 26 mm) and a long AL group (AL ≥ 26 mm). The inclusion criteria were as follows: (1) age between 18 and 35 years; (2) stable refractive state, and degree change within ± 0.5°diopters (D) in the recent 2 years; and (3) normal-range IOP and normal optic nerves. The exclusion criteria were as follows: (1) inability to obtain good-quality images due to poor fixation; (2) a history of intraocular surgery or trauma; (3) systemic diseases such as diabetes mellitus and hypertension that may affect ocular hemodynamics; (4) other ocular diseases such as glaucoma or severe xerophthalmia; (5) ocular media opacity; and (6) myopic maculopathy greater than Category 1 (tessellated fundus) based on the International Photographic Classification and Grading System for myopic maculopathy (16).

### Optical coherence tomography angiography

Optical coherence tomography angiography images were obtained using a Cirrus HD-OCT 5000 angiography system (Carl Zeiss Meditec Inc., Dublin, CA, United States). It uses Doppler technology and optical microangiography technology to observe the vascular structure and blood flow velocity of the fundus and reduces motion artifacts using a patented retinal tracking technology called FastTrac™. It provides three-dimensional fundus angiography, which can capture images and scan at a faster rate (17). The automatic layered technology can automatically detect different layers of the retina. However, its delamination does not conform to the standard definition of fundus vascular delamination, where the boundary of the superficial layer is set to < 3 mm of the internal limiting membrane and < 15 mm of the inner plexiform layer (18).

**FIGURE 1:**
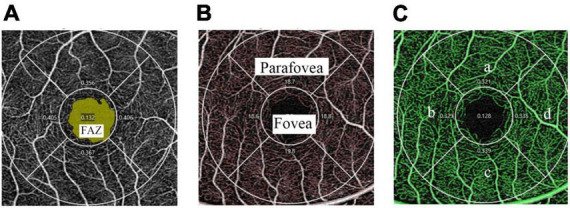
Superficial vessel density images of OCTA in a 3 × 3 mm scan mode. **(A)** The FAZ area (marked as yellow) is automatically recognized by the software. **(B)** The skeletonized vasculature (marked as red) is automatically recognized to calculate VLD and the fovea and parafovea areas are marked separately. **(C)** The perfused vasculature (marked as green) is automatically recognized to calculate PD and is divided into four quadrants: superior (a), temporal (b), inferior (c), and nasal (d).

We obtained a 3 × 3 mm macular scan from each eye. Each scan was captured for the best image to obtain the vessel length density (VLD) and perfusion density (PD) in the superficial layer OCTA images. Superficial vessel density images of OCTA are presented in Figure 1, where VLD was defined as the total length of the skeletonized perfused vasculature per unit area (Figure 1B). PD was defined as the total area of perfused vasculature per unit area (Figure 1C). The “parafovea” was defined as an annulus centered on the fovea with inner and outer ring diameters of 1 and 3 mm. The exclusion criteria for the OCT angiography scan were as follows: (1) signal strength < 6, (2) poor image quality caused by poor fixation, and (3) fovea not in the center of the scanned area. The magnification of imaging the fundus have been corrected in myopia because of the elongation of the eye in the present study (19).

### Statistical analysis

Statistical analysis was performed using the SPSS software (version 25.0; IBM Corp., Armonk, NY, United States). A generalized estimated equation was used to adjust for the correlation between the two eyes of the same subject. Data distributions were tested for normality using the Kolmogorov–Smirnov test. Mann–Whitney *U* test and independent-sample *t*-test were used to compare the means between the two groups. Spearman’s correlation analysis and Pearson’s correlation analysis were used to analyze the correlation between vascular density and ocular factors including AL and SE. Multiple regression analysis were used to analyze the relationship between retinal vascular density and visual quality. Two binary logistic regression models were used to evaluate the vessel density as a risk factor for BCVA by categorizing the patients into two groups: −0.079 LogMAR (20/16 Snellen equivalent) or worse; better than −0.079 LogMAR. Quantitative data were expressed as mean ± standard deviation (SD), and parameters that did not follow the normal distribution were expressed as medians and quartiles. Statistical significance was set at *P* < 0.05.

## Results

### Basic patient characteristics

In total, 199 eyes were included in the analysis. A total of 109 eyes (34 men and 23 women) were included in the medium AL group. The mean age was 20.00 years (19.00, 25.00). The mean SE was −4.57 ± 1.37 D, and the mean AL was 25.40 mm (25.16, 25.67). Ninety eyes (24 men and 28 women) were included in the long AL group. The mean age was 20.00 years (18.75, 21.00), the mean SE was −7.19 ± 2.13 D and the mean AL was 26.60 mm (26.29, 27.11). As expected, the axial length, SE, UCVA, and BCVA were significantly different between the two groups (Table 1, all *P* < 0.001). No significant differences were found in sex, age, or pupil diameter between the groups (Table 1).

**TABLE 1:** Demographic and clinical characteristics between medium AL group and long AL group.

Parameters	Medium AL group	Long AL group	*P*-value
*N*	109	90	–
Sex (male:female)	57/52	48/42	0.884
Age (years)	20.00 (19.00, 25.00)	20.00 (18.75, 21.00)	0.058
SE (D)	−4.57 ± 1.37	−7.19 ± 2.13	**<0.001**
AL (mm)	25.40 (25.16, 25.67)	26.60 (26.29, 27.12)	**<0.001**
UCVA (logMAR)	1.00 (0.70, 1.10)	1.10 (1.00, 1.22)	**<0.001**
BCVA (logMAR)	−0.18 (−0.18, −0.08)	−0.08 (−0.18, −0.08)	**<0.001**
Pupil diameter (mm)	4.92 ± 0.66	5.05 ± 0.63	0.147

Data are shown as mean ± standard deviation, medians and quartiles, or *n* values; Significant *P*-values are indicated in bold.

### Optical coherence tomography angiography parameters

**FIGURE 2:**
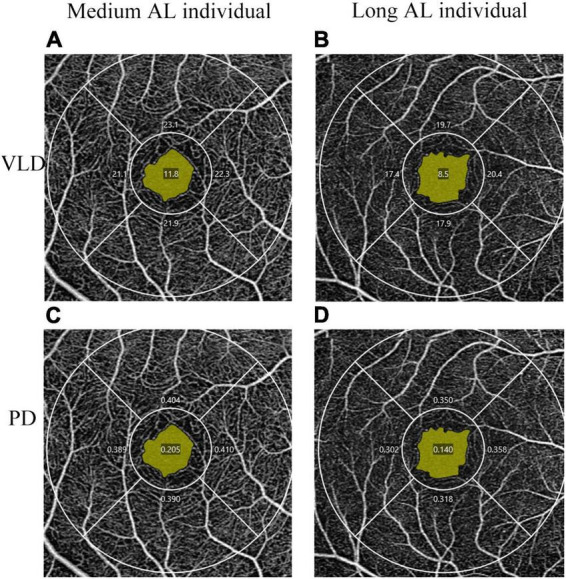
OCTA images of VLD **(A,B)** and PD **(C,D)** between medium and long axial length individuals.

There were significant differences between the two groups in terms of the inferior VLD and parafovea PD (Table 2). The FAZ area also showed a significant difference between the two groups (*P* = 0.011). No significant differences were found between groups for the signal strength. OCTA images between medium and long axial length individuals are also shown in Figure 2 and it’s clear that the vessel density is lower in eyes with longer axial length.

**TABLE 2:** Macular vessel density and FAZ parameters between medium AL group and long AL group.

Parameters	Medium AL group	Long AL group	*P*-value
Fovea VLD (mm^–1^)	10.10 (8.80, 11.65)	10.70 (9.00, 12.73)	0.059
Parafovea VLD (mm^–1^)	21.40 (20.50, 22.10)	21.15 (20.08, 21.88)	0.258
Whole VLD (mm^–1^)	20.10 (19.20, 20.80)	20.00 (18.88, 20.83)	0.595
Temporal VLD (mm^–1^)	21.20 (20.10, 22.15)	21.00 (20.10, 22.10)	0.433
Superior VLD (mm^–1^)	21.40 (20.10, 22.25)	21.40 (19.85, 22.23)	0.965
Nasal VLD (mm^–1^)	21.80 (20.55, 22.50)	21.60 (20.58, 22.63)	0.537
Inferior VLD (mm^–1^)	21.20 (20.25, 22.00)	20.65 (19.48, 21.70)	**0.013**
Fovea PD	0.170 (0.149, 0.198)	0.178 (0.154, 0.216)	0.158
Parafovea PD	0.382 (0.369, 0.395)	0.374 (0.360, 0.389)	**0.036**
Whole PD	0.358 (0.342, 0.370)	0.354 (0.337, 0.367)	0.190
Temporal PD	0.381 (0.362, 0.397)	0.376 (0.357, 0.393)	0.199
Superior PD	0.381 (0.368, 0.397)	0.382 (0.356, 0.396)	0.461
Nasal PD	0.388 (0.370, 0.405)	0.383 (0.361, 0.398)	0.083
Inferior PD	0.380 (0.362, 0.396)	0.373 (0.355, 0.390)	0.064
FAZ area (mm^2^)	0.28 ± 0.09	0.25 ± 0.08	**0.011**
FAZ perimeter (mm)	2.21 ± 0.38	2.11 ± 0.39	0.075
FAZ circularity	0.70 ± 0.08	0.69 ± 0.10	0.844
Signal strength	9.21 ± 0.97	9.06 ± 1.09	0.252

Data are shown as medians and quartiles or mean ± standard deviation; Significant *P*-values are indicated in bold.

### Correlation between vessel density and axial length, spherical correction

**FIGURE 3:**
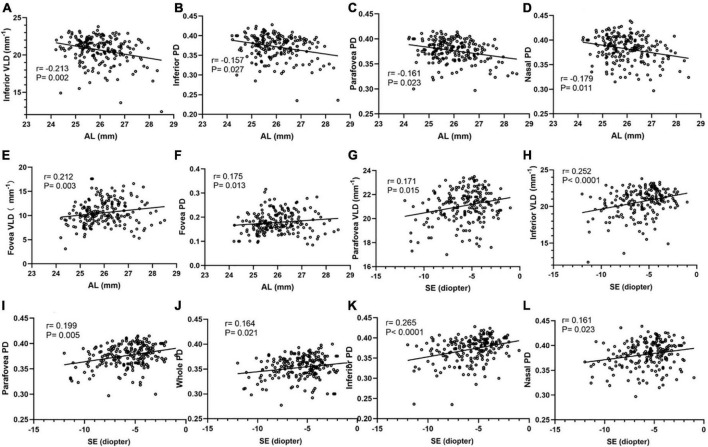
Correlation analyses between macular vessel density and AL, SE in different areas of two calculation methods. The inferior VLD **(A)**, inferior PD **(B)**, parafovea PD **(C)**, and nasal PD **(D)** show a negative correlation with AL. The fovea VLD **(E)** and fovea PD **(F)** show a positive correlation with AL. The parafovea VLD **(G)**, inferior VLD **(H)**, parafovea PD **(I)**, whole PD **(J)**, inferior PD **(K)**, nasal PD **(L)** show a positive correlation with SE.

The correlation analyses between macular vessel density and AL and SE in different areas of the two calculation methods are shown in Figure 3. Spearman’s correlation test showed a significant negative association between AL and inferior VLD, parafovea PD, inferior PD, and nasal PD. The vessel densities in the superior and temporal regions were not corrected for AL. Fovea vessel density was positively correlated with AL (*r* = 0.175, *P* = 0.013). Spearman’s correlation test showed a significant negative association between SE and the parafovea VLD, inferior VLD, parafovea PD, inferior PD, nasal PD, and whole PD, but no fovea, temporal, and superior regions.

### Correlation between fovea avascular zone and axial length, spherical correction

**FIGURE 4:**
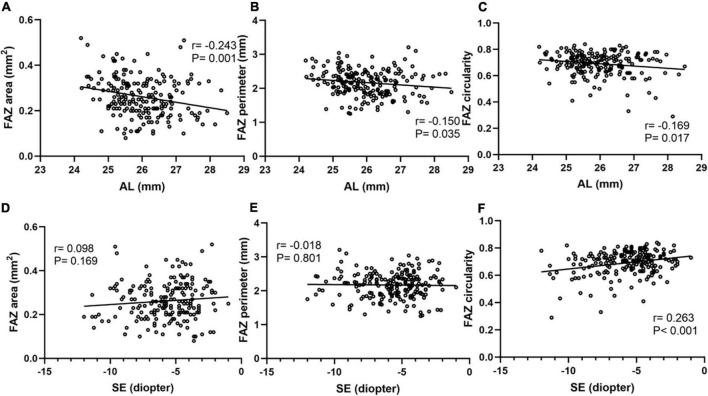
Correlation analyses between FAZ parameters and AL, SE. FAZ area **(A)**, perimeter **(B)** and circularity **(C)** all showed a significant negative association with AL. FAZ area **(D)** and perimeter **(E)** showed no obvious correlation with SE. FAZ circularity **(F)** showed a significant positive association with SE.

Correlation analyses were performed using Pearson’s correlation test in Figure 4. FAZ area, perimeter and circularity all showed a significant negative association with AL using Pearson’s correlation analysis. FAZ circularity was positively correlated with SE (*r* = 0.263, *P* < 0.001). Additionally, a partial correlation analysis was performed to control the confounding factor of signal strength. FAZ area and perimeter still showed a significant negative association with AL (*r* = −0.227, *P* = 0.001; *r* = −0.156, *P* = 0.029). FAZ circularity also showed a positively correlation with SE after adjusting signal strength (*r* = 0.219, *P* = 0.002).

### Correlation between vessel density and visual quality

**FIGURE 5:**
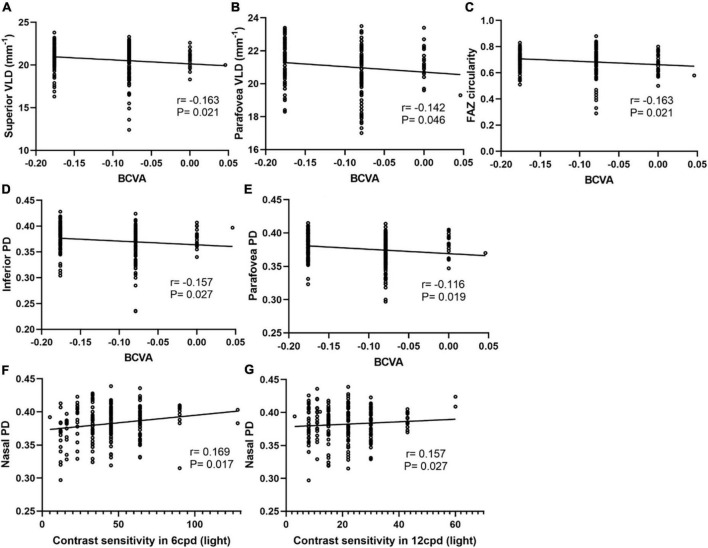
Correlation analyses between macular vessel density and BCVA, CSF in different areas of two calculation methods. The superior VLD **(A)**, parafovea VLD **(B)**, FAZ circularity **(C)**, inferior PD **(D)**, and parafovea PD **(E)** show a negative correlation with BCVA. Nasal PD shows a positive correlation with CSF with bright around at 6 cpd **(F)** and 12 cpd **(G)**.

Although no significant differences in CSF and HOAs were found between these two groups, there was a significant positive association between nasal PD and CSF with bright around 6 and 12 cycles per degree (cpd) (Figure 5). The parameters that were significantly correlated with the CSF were included in the multiple regression analysis. The nasal PD still showed a significant association with CSF in bright 6 cpd (β = 3.036, *P* = 0.036) and a borderline significance with CSF in bright 12 cpd (β = 2.567, *P* = 0.069) after adjusting for age and SE.

The difference in BCVA between the two groups was statistically significant (*P* < 0.001), and BCVA had a significant positive association with parafovea VLD, superior VLD, parafovea PD, inferior PD, nasal PD and FOZ circularity (Figure 5). Further confirmation multivariate binary logistic regression analysis was used to evaluate factors associated with BCVA and performed by stratifying BCVA into a binary variable at a selected cutoff point. The results showed that higher parafovea PD and shorter AL were significantly associated with better BCVA (Table 3).

**TABLE 3:** Multivariate logistic regression analysis to evaluate PD as the risk factor for BCVA.

Influence factors	Odds ratio (95% CI)	*P*-value
Parafovea PD	1.020 (1.003–1.037)	**0.020**
AL (mm)	0.581 (0.398–0.848)	**0.005**
Age (years)	0.942 (0.869–1.022)	0.151
Signal strength	1.172 (0.849–1.617)	0.334

Subjects were categorized into two subgroups based on BCVA in the binary logistic regression: one subgroup had subjects with a BCVA of −0.079 (20/16) or worse and the other subgroup had subjects with a BCVA better than −0.079 (20/16). CI, confidence interval; Significant *P*-values are indicated in bold.

## Discussion

In this study, we utilized OCTA to measure the distribution of superficial retinal vascular density in eyes with long and medium AL. We also evaluated its relationship with AL and SE, and its impact on BCVA, CSF, and HOAs. We found that parafovea vascular density tended to be lower in myopic patients with a longer eye axis, and also showed a significant positive association with SE. In addition, the decrease in vessel density could worsen BCVA and lower CSF at high spatial frequencies.

We found that the PD of the parafovea region and VLD of the inferior region decreased more significantly in the long AL group than in the medium AL group, but there was no significant change in foveal VLD and PD. Shi et al. (10) also found that high myopes showed greater loss of macular vessel density over time globally, especially in the inner-inferior, inner-temporal, and outer-temporal sectors. The way blood vessel density is quantified can be explained as VLD in this study. The result of our study is different from a study which suggest that patients with high myopia have no significant changes in the parafovea vascular density (20). However, more studies have suggested the changes in vascular density in the parafovea area (21–23).

Correlation analysis showed a significant negative association between AL and parafoveal vessel density and between SE and parafoveal vessel density. Previous studies found that retinal vessel density decreased in high myopia (24), which may be due to the increase in axial length (9, 10, 25). Some studies also found a significant correlation between retinal vascular density and SE (26, 27) which is concordant with our study. We speculate that, along with the increase in refractive error and axial length, the eyeball mechanically stretches the retinal tissue, causing retinal and choroidal thinning, which may cause a decline in the retinal vascular network (28). Retinal vascular endothelial cells and retinal pigment epithelium cells that produce vascular endothelial growth factors, which play an important role in stimulating vessel growth, may also contribute to the loss of capillary network, and VEGF has already been found to have a negative correlation with axial length (29). However, the exact mechanism remains unclear.

Our results, consistent with those of previous studies (9, 21, 30), show that the change in vessel density in the inferior and nasal sectors is more obvious. The results of this study can be explained by the loss of the nerve fiber layer, which often occurs in peripapillary atrophy on the temporal side. According to previous studies, there is a strong correlation between retinal inner layer thickness and blood perfusion (30), and significant reductions in vessel density were associated with lengthening of the AL and reduction in the retinal nerve fiber layer and ganglion cell complex thickness (31). A decrease in retinal thickness may lead to a decrease in oxygen and nutrient demand, which leads to a decrease in blood perfusion density. This means that mechanical stretching of the eye wall reduces its thickness and causes a secondary lower demand of tissues for oxygen, but another theory suggests that reduced circulation leads to secondary thinning of the tissues (28). Further investigation is required to determine which theory is more acceptable.

We found that the FAZ area showed a significant negative association with AL. This finding is in agreement with the conclusions of a recent study (32). The change in FAZ area in myopia remains a matter of controversy. Li et al. (23) reported that the FAZ area did not show a significant difference between highly myopic eyes and control group eyes, but others suggest that the FAZ area was smaller in people with high myopia and longer AL (21, 33). The negative correlation of FAZ area with axial length observed in the correlation analysis of this study may be due to the lower optical magnification in eyes with longer axial length (33), while FAZ area and perimeter did not show correlation with SE. The positive correlation of foveal vessel density with AL may also be due to this factor. We also found a correlation between FAZ circularity and SE. The FAZ circularity was calculated as the perimeter of the FAZ divided by the standard circular perimeter of an equal area. According to our results, the smaller the diopters become, the closer they become to a perfect circle.

We also found that a decrease in vessel density could contribute to worse BCVA. Previous studies have found a correlation between retinal vessel density and visual acuity (11), which shows the importance of changes in the vascular structure to the patient’s vision.

Furthermore, we found that retinal vessel density could be related to CSF. Liu et al. (15) also found the correlation between CSF and the changes of high myopia fundus. For high myopia, loss of contrast sensitivity at higher spatial frequencies was observed, which may be due to retinal function disturbances, which may occur at an early stage even before the onset of retinal pathological events (14). Contrast sensitivity can be affected by the function of photoreceptor cells (34). The arrangement of the photoreceptors in high myopia is affected by excessive stretch in the posterior pole, which may lead to a subnormal visual function. In high myopia, cones in the nasal hemiretina are aligned toward the optic nerve, whereas they are aligned toward the center of the exit pupil in the temporal hemiretina. This discrepancy in receptor alignment is directly associated with the axial length. The specific types of contrast sensitivity changes may be a result of specific neural loss (14). Our results show a significant positive association between nasal PD and contrast sensitivity to luminance-defined, high spatial frequency gratings, which also supports this theory. Zheng et al. (35) demonstrated that the macular sensitivity was correlated with deep retinal PD but not with superficial retinal PD in highly myopic eyes with myopic macular degeneration by using microperimetry. They suggested that this might be due to the morphologic degeneration of photoreceptors in the deep retinal capillary plexus. Wang et al. (36) also found the correlation between decreased macular sensitivity and morphologic degeneration of photoreceptors in high myopes using microperimetry and adaptive optics. Hoffmann et al. (37) measured visual function and retinal morphologic parameters in patients with age-related macular degeneration and found correlations between contrast sensitivity and retinal anatomic features. They suggested that CSF may serve as a better biomarker in early disease stages. Moreover, our research found that contrast sensitivity was correlated with measures of paranasal structures, but not with foveal structures. We speculate that functional vision may be affected earlier than foveal microstructure, which is in agreement with a previously reported finding (38).

Our results showed that there was no significant correlation between vascular density in the macular area and total high-order aberrations, and the total high-order aberrations among the four groups were not statistically significant. However, some studies have indicated that with an increase in the degree of myopia, high-order aberrations would also increase (39). Our conclusion that there was no statistical significance may be due to the influence of elongated AL on intraocular high-order aberrations, thus playing a certain compensating role in corneal high-order aberrations.

Current studies suggest that retinal vascular density can be affected by age; with increasing age (40), the self-regulation ability of retinal blood vessels decreases gradually (41), and some publications indicate that the FAZ area can also be affected by age (42, 43); therefore, to exclude the influence of age, the participants we enrolled were young adults. With age, the self-regulation ability of retinal blood vessels gradually decreases, and younger subjects generally tend to fixate better (44).

The study has several limitations. First, it was a cross-sectional study. Future longitudinal studies are needed to analyze the sequence of the choroid and retinal structure and visual function changes to elucidate the mechanism of visual impairment in high myopia. Second, we only evaluated the parameters in the macular area without the optic disc area, which will be the focus of our future study.

## Conclusion

In conclusion, we confirmed that the superficial retinal vascular density of young myopic adults was reduced with lower SE and longer AL in the parafoveal area, especially in the inferior sector. An eye with a long AL has a smaller FAZ, and myopia decreases the FAZ circularity index. The decrease in vessel density in young myopic adults could contribute to worse BCVA and may be correlated with lower contrast sensitivity at mid to high spatial frequencies, but not with HOAs.

## Data availability statement

The raw data supporting the conclusions of this article will be made available by the authors, without undue reservation.

## Ethics statement

The studies involving human participants were reviewed and approved by the Ethics Committee of Shengjing Hospital of China Medical University (2020PS720K). The patients/participants provided their written informed consent to participate in this study.

## Author contributions

XW collected and analyzed the data and wrote the manuscript. LX designed and supervised the study. Both authors contributed to the revision of the manuscript.
